# Gastroenterology Curriculum in the Canadian Medical School System

**DOI:** 10.1155/2017/8538974

**Published:** 2017-04-06

**Authors:** ThucNhi Tran Dang, Clarence Wong, Lana Bistritz

**Affiliations:** Faculty of Dentistry and Medicine, University of Alberta, Edmonton, AB, Canada

## Abstract

*Background and Purpose.* Gastroenterology is a diverse subspecialty that covers a wide array of topics. The preclinical gastroenterology curriculum is often the only formal training that medical students receive prior to becoming residents. There is no Canadian consensus on learning objectives or instructional methods and a general lack of awareness of curriculum at other institutions. This results in variable background knowledge for residents and lack of guidance for course development.* Objectives.* (1) Elucidate gastroenterology topics being taught at the preclinical level. (2) Determine instructional methods employed to teach gastroenterology content.* Results*. A curriculum map of gastroenterology topics was constructed from 10 of the medical schools that responded. Topics often not taught included pediatric GI diseases, surgery and trauma, food allergies/intolerances, and obesity. Gastroenterology was taught primarily by gastroenterologists and surgeons. Didactic and small group teaching was the most employed teaching method.* Conclusion.* This study is the first step in examining the Canadian gastroenterology curriculum at a preclinical level. The data can be used to inform curriculum development so that topics generally lacking are better incorporated in the curriculum. The study can also be used as a guide for further curriculum design and alignment across the country.

## 1. Background

Gastroenterology is a diverse subspecialty that covers a wide array of topics ranging from functional abdominal pain to gastrointestinal (GI) malignancies. Gastrointestinal disorders are seen at all levels of care including primary care, inpatient medicine, and surgery. A recent study showed that gastrointestinal complaints make up a significant burden of disease and accounted for over 30 million outpatient clinic visits alone in the United States in 2009 [1]. The preclinical gastroenterology curriculum is often the only formal GI training that medical students receive prior to becoming residents and is the main opportunity for students to develop fundamental knowledge of GI diseases.

Over the past few decades, Canadian medical schools have moved from didactic approaches to teaching towards more interactive methods such as small group sessions, simulated patient cases, peer teaching, computer-based learning, and other interactive methods of education [2–4]. Most Canadian medical schools have embraced this approach and incorporated it into the preclinical curriculum. Despite this change in teaching methodology and the diverse topics in GI, there is no national consensus on GI-specific learning objectives or instructional methods for the gastroenterology curriculum in Canada. The Medical Council of Canada has published objectives for the LMCC examinations [5], but these are now over a decade old and are not intended to be used as objectives for undergraduate medical curriculum development [6]. These objectives are not specialty-based which may make them difficult to use for course coordinators. For example, colon cancer screening is nested under “Periodic Health Examination” in the MCC objectives document. At the postgraduate level, significant work has been done to move to a competence based medical education framework, but the learning outcomes even at this level are still not established and would not be applicable to an undergraduate medical setting [7]. This culminates in uncertainty regarding what content domains should be included in the undergraduate medical setting.

Given the lack of consistent guidance regarding gastroenterology objectives to be covered at the undergraduate level, the possibility for variability among Canadian medical student curricula exists. This contributes to the development of educational silos and may result in junior residents with inconsistent GI knowledge at the onset of residency training. Consistent objectives for undergraduate GI education would ensure consistent knowledge and skills for all Canadian medical students, guide course coordinators and instructors in curriculum and learning session planning, and enable medical schools to compare their curricula to a benchmark for program evaluation, and possibly accreditation. As a first step towards this ultimate goal, we sought to determine the current range of undergraduate gastroenterology education in Canada, including content domains and instructional methods.

## 2. Methods

A cross-sectional survey design was used for this study. Ethics approval was obtained from the University of Alberta Research Ethics Board. A survey instrument of gastroenterology content domains, teaching methods, and assessment tools was developed by two content experts (LB, CW) with significant undergraduate education experience and involvement in the undergraduate gastroenterology curriculum at the University of Alberta. In addition to an extensive list of gastroenterology topics, we also included nongastroenterology topics (i.e., dysmenorrhea, pneumonia) as a quality measure to ensure respondents were accurately answering the survey. This survey was piloted internally to two other gastroenterologists from the University of Alberta and subsequently to two external gastroenterologists involved in organizing the gastroenterology curriculum at other institutions for review. After survey modification based on the pilot testing, the final survey instrument (full version of the final survey included in Supplementary Material available online at https://doi.org/10.1155/2017/8538974) was sent electronically to the gastroenterology preclinical curriculum coordinators at all 17 Canadian medical schools in October 2014. A reminder was sent 2–4 weeks after initial contact if no response was received and again in 6 months after initial contact.

Responses were coded by institution. Data entry and descriptive statistics were performed using Microsoft Office 365 ProPlus Excel v.16.0.6741.2071. A curriculum map was developed as a visual representation of curriculum commonalities and gaps between Canadian medical schools.

## 3. Results

A total of 10 schools with annual entering class sizes ranging from 100 (Queen's University, University of Saskatchewan) to 288 (University of British Columbia) participated in the survey (Table 1) (response rate 10/17, 58.8%). Nine of the ten schools were English-speaking programs while the remaining school was a French-speaking program. None of the schools acknowledged teaching dysmenorrhea or pneumonia in our survey.

### 3.1. Curriculum Assessment

**Figure 1:**
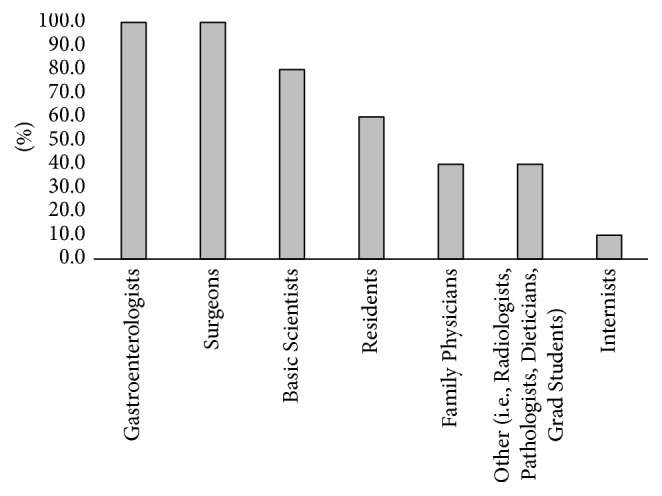
Specialists involved in the undergraduate medical curriculum at Canadian medical schools.

**Figure 2:**
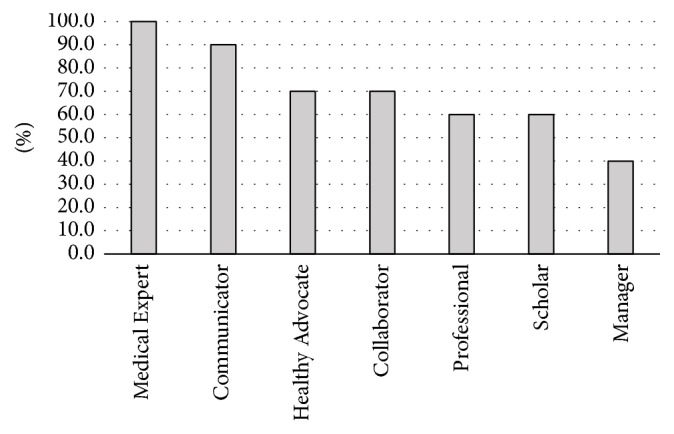
CanMEDs roles formally taught in the undergraduate gastroenterology curriculum.

Eight of the schools' medical curriculum followed the traditional four-year program with two years of preclinical training and two years of clerkship rotations. The remaining two institutions followed a curriculum that extended over three years and consisted of two years of preclinical training and one year of clerkship rotation. All of the schools incorporated a formal GI course in the preclinical curriculum while additional six schools incorporated a formal GI course in the clerkship rotation. According to the survey, both gastroenterologists (100%) and surgeons (100%) were the specialists primarily involved in teaching the GI curriculum (Figure 1). Sixty-percent of the institutions involved residents in teaching the GI curriculum. All of the Canadian medical institutions incorporated at least one of the CanMEDs roles into the formal GI curriculum (Figure 2).

**Figure 3:**
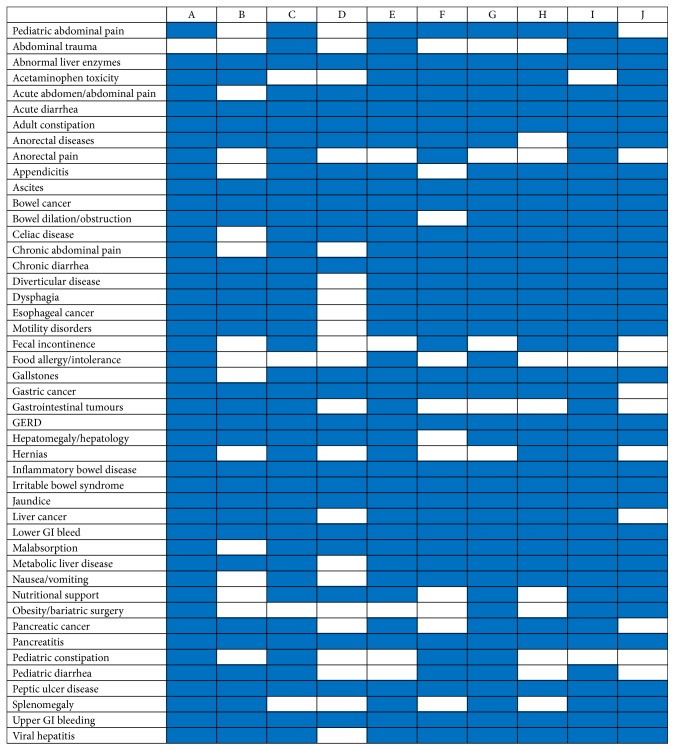
Gastroenterology curriculum map.

A curriculum map of the content areas taught was compiled from the survey responses (Figure 3). Responses gathered from the Canadian medical schools show that there is a heterogeneous curriculum that is taught across the country. However, topics that were universally taught (>80%) included liver topics (i.e., viral hepatitis, metabolic liver diseases, hepatomegaly, etc.), acute/chronic diarrhea, autoimmune GI diseases (i.e., inflammatory bowel disease and Celiac disease), upper/lower GI bleeding, pancreatobiliary disease, esophageal and bowel neoplasms, and dysphagia and motility disorders.

Surgical topics such as abdominal trauma (40%), anorectal pain (40%), fecal incontinence (50%), gastrointestinal tumours (50%), hernias (50%), and obesity/bariatric surgeries (40%) were taught at half or less than half of the medical institutions. Other topics that were found to be lacking in the GI curriculum in Canadian medical schools include food allergies and intolerances (30%), pediatric constipation (40%), and pediatric diarrhea (50%).

### 3.2. Teaching Methods

**Figure 4:**
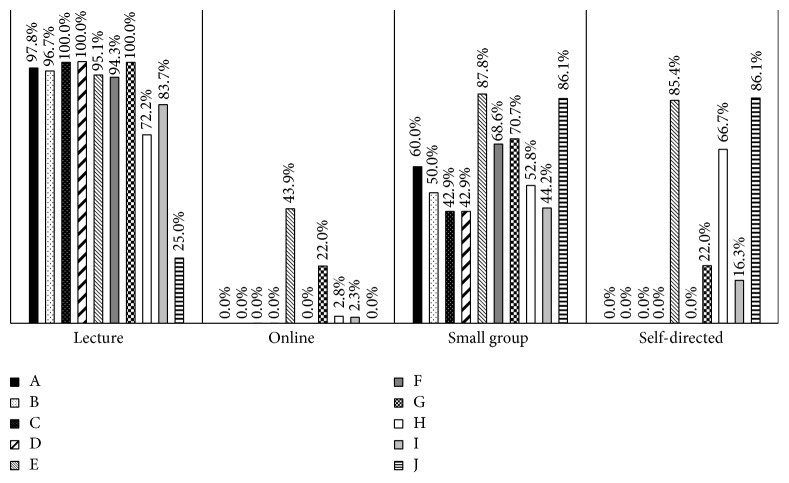
Teaching methods by Canadian medical schools.

**Figure 5:**
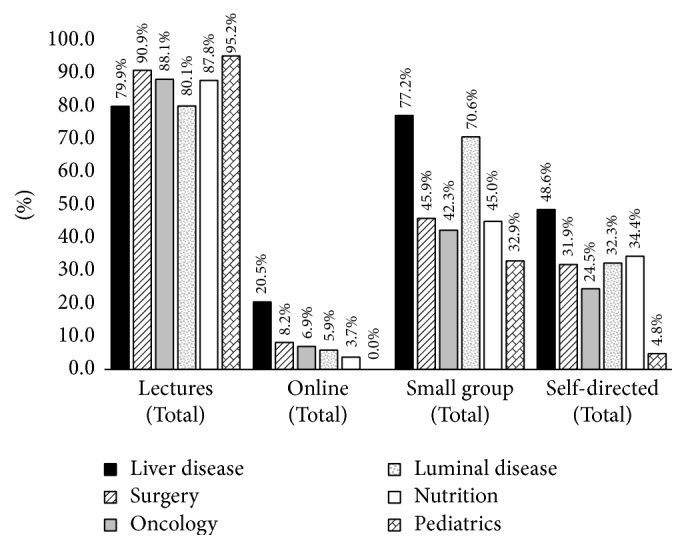
Teaching methods by gastrointestinal topics.

Despite the inclusion of problem-based teaching and small group sessions, didactic lectures remained the primary teaching method employed by medical schools (Figure 4). Only one school (J) used alternative instructional approaches more than didactic lectures. Small group sessions were the second most frequently used method of instruction, followed by self-directed learning, then online modules/resources. When stratified by themes, liver topics employed the most diverse teaching methods, followed by luminal disease, then surgery (Figure 5). Pediatric topics were taught primarily didactically (95%) with relatively little small groups (33%), self-directed learning (5%), or online modules/resources (0%).

### 3.3. Assessments

**Figure 6:**
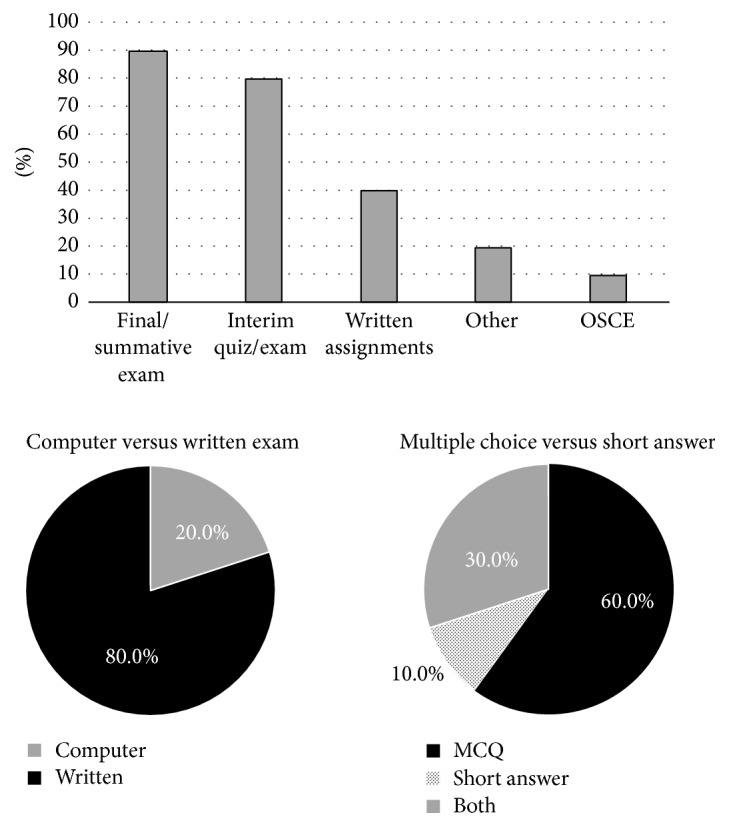


The traditional model of final/summative examinations (90%) remained the most popular method of evaluating medical students' GI-specific knowledge. Only one institution used an objective structured clinical exam (OSCE) to assess medical students on their GI knowledge (10%). Despite technologic advances, most institutions still used handwritten exams for their assessments. Additionally, most schools used only multiple choice questions (MCQ) based exams (60%), followed by combined MCQ and short answer (30%), with only 10% using short answer-only (Figure 6).

## 4. Discussion

As previously mentioned, gastrointestinal complaints and disorders carry a significant burden of disease and accounted for over 30 million outpatient clinic visits [1]. According to a Canadian audit examining the wait times for gastroenterology subspecialty care, the median wait time was three months following referral. Even with abnormal test results, patients waited almost two months following referral. With alarm features noted in consultations, the median wait times were improved at 43 days compared to 82 days for consultations without any alarm features [8]. In a separate study Yu et al. showed that the average wait time for endoscopic evaluation was 229 days; for those who were determined on endoscopy to have serious diagnosis, the average wait time for endoscopic evaluation was 115 days [9], presumably due to the recognition of alarm symptoms. These studies highlight the importance of primary care physicians in both triaging gastrointestinal complaints (i.e., recognition of alarm features) as well as their role in managing gastrointestinal complaints until subspecialty care can be obtained.

While the data is limited, there are studies which suggest that primary care physicians have specific knowledge gaps in both diagnosis and chronic management of gastrointestinal diseases. Most of the evidence centers around primary care physicians' discomfort in diagnosing and managing viral hepatitis and hepatocellular carcinoma [10–13]. However, there are studies to suggest even common GI ailments such as irritable bowel syndrome [14, 15], Helicobacter pylori [16], and gastroesophageal reflux disease [17] can cause confusion and angst among primary care physicians.

The undergraduate GI medical curriculum in medical school is often the only direct exposure students have to gastroenterology subspecialists prior to entering residency, especially in the case of primary care. As a result, the undergraduate GI curriculum is frequently where fundamental knowledge of the pathophysiology, diagnosis, and management of GI disease processes is acquired. Ensuring that core gastroenterology concepts are learned and competencies are reached by medical students across the country will be necessary to ensure future primary care physicians have the core knowledge and skills required to manage the spectrum of patients with GI diseases. Although there have been a number of studies examining the undergraduate curriculum in other subspecialties such as dermatology, emergency medicine, and oncology [18–21], this is the first study that describes the current state of the undergraduate gastroenterology curriculum across Canada. It is encouraging that of the medical schools that responded all had formal GI instruction in the preclerkship curriculum and some schools even incorporated GI in their clinical clerkship. However, the gastroenterology curriculum across the country is fairly heterogeneous. Topics which are taught in less than half of the medical schools included surgical topics, food allergies/intolerances, pediatric GI diseases, and obesity/bariatric management. These topics are important for primary care physicians, general internists, and surgeons and are crucial for gastroenterologists. As the Royal College of Physician and Surgeons moves towards a competency-based model, these deficiencies at the undergraduate curriculum may translate to more notable knowledge gaps at the postgraduate and fellowship levels.

There were some limitations in our study. Because the study is based on a survey instrument, responses gathered from institutions were influenced by both response rate and response bias [22, 23]. Given that the survey instrument was developed internally, the GI topics and teaching modalities included in the survey may have been biased by the institution's own curriculum and curriculum layout; however, this was partially offset by piloting the survey instrument to two external gastroenterologists involved in curriculum development at other institutions. Since the survey was only distributed to the coordinators of the gastroenterology block, it is unable to capture whether certain topics were taught in a separate part of the undergraduate medical curriculum (i.e., whether pediatric GI diseases were covered in a pediatric block or rotation).

## 5. Conclusions and Future Directions

This was the first study examining the gastroenterology curriculum at the undergraduate medical curriculum. Future studies should attempt to elicit responses from the remaining Canadian medical institutions and correlate topics taught in the curriculum and medical students' perceived comfort with those topics as well as objective measures of their GI knowledge such as their performance on GI questions on Medical Council of Canada Qualifying Examination Parts I and II. The curriculum map can also be used as a guide for individual institutions to tailor future curriculum development to address curriculum gaps.

## Supplementary Material

The final survey consists of a survey instrument covering gastroenterology topics taught at the undergraduate medical curriculum, the teaching and evaluation methods employed, and CanMED roles.

## Figures and Tables

**Table 1:** Medical schools that participated in the survey.

University of Alberta	
University of British Columbia	
University of Calgary	
University of Manitoba	
McMaster University	
Queen's University	
University of Saskatchewan	
University of Sherbrooke	
University of Toronto	
Western University	
