# Supplementary material for: Gastroenterology Curriculum in the Canadian Medical School System
Source: Can J Gastroenterol Hepatol. 2017 Apr 6;2017:8538974. doi: 10.1155/2017/8538974 (PMC5397633; doi:10.1155/2017/8538974)
Supplement: Supplementary file 1 — The final survey consists of a survey instrument covering gastroenterology topics taught at the undergraduate medical curriculum, the teaching and evaluation methods employed, and CanMED roles. [file 8538974.f1.pdf]

UNIVERSITY OF ALBERTA
FACULTY OF MEDICINE & DENTISTRY
Department of Medicine

Undergraduate Gastroenterology Curriculum Survey 2014

1. Select your University:

2. How is the undergraduate Gastroenterology taught (choose all that apply)?

- ☐ Pre-clinical curriculum
- ☐ Clinical curriculum (i.e. clerkship)
- ☐ Other

3. Is there a Gastroenterology undergraduate course organized and taught in a discrete course or blcok?

- ◯ Yes (go to questions 4)
- ◯ No (go to question 5)

---

4. Who coordinates / organizes the Gastroenterology pre-clinical course?

- ◯ Gastroenterologist
- ◯ Other. Please specify:

5. Who is involved in teaching the pre-clinical course (choose all that apply)?

- ☐ Basic scientists (i.e. anatomy, physiology, biochemistry, pharmacology, etc.)
- ☐ Family physicians
- ☐ Gastroenterologists
- ☐ Internists (other)
- ☐ Surgeons
- ☐ Residents
- ☐ Other

---

6. If there is a specific Gastroenterology course, in what year of medical school is it delivered (choose all that apply)?

- ☐ Year 1
- ☐ Year 2
- ☐ Year 3
- ☐ Year 4

7. How long is the Gastroenterology course/curriculum?

◯ Less than 4 weeks

◯ 4 to 8 weeks

◯ 8 to 12 weeks

◯ More than 12 weeks

8. What instructional methods are employed in the undergraduate Gastroenterology curriculum at your university (choose all that apply)?

☐ Didactic lecture

☐ Small group learning

☐ Problem-based learning

☐ Clinical exposure

☐ Physical examination / bedside clinical teaching sessions

☐ Laboratory sessions (i.e. anatomy)

9. Is the clinical Gastroenterology taught separately from basic science content (i.e. pathophysiology, anatomy, pharmacology, etc.) **or integrated in a systems-based approach** (i.e. integrated clinical and basic science content within the same lecture / course)**?**

◯ Basic science separate from clinical Gastroenterology

◯ Integrated approach

10. Are Gastroenterology topics taught based on an approach to a symptom or an approach to a specific disease?

◯ Symptom-based approach (i.e. approach to dysphagia)

◯ Disease-based approach (i.e. approach to gastroesophageal reflux disease)

◯ Both

11. Do you use the Medical Council of Canada objectives in planning your curriculum?

◯ Yes

◯ No

---

12. Please indicate if the following topics are covered in your undergraduate medical curriculum (check all that apply). If covered, please indicate in which format these topics are discussed (check all that apply):

☐ ***Abdominal pain in children***	☐ Lecture	☐ Small Group
	☐ Online	☐ Self-directed
☐ ***Abdominal trauma***	☐ Lecture	☐ Small Group
	☐ Online	☐ Self-directed
☐ ***Abnormal liver enzymes***	☐ Lecture	☐ Small Group
	☐ Online	☐ Self-directed

☐ ***Acetomenophen toxicity***	☐ Lecture	☐ Small Group
	☐ Online	☐ Self-directed
☐ ***Acute abdominal pain / acute abdomen***	☐ Lecture	☐ Small Group
	☐ Online	☐ Self-directed
☐ ***Acute diarrhea***	☐ Lecture	☐ Small Group
	☐ Online	☐ Self-directed
☐ ***Adult constipation***	☐ Lecture	☐ Small Group
	☐ Online	☐ Self-directed
☐ ***Ano-rectal disease (i.e. fissures, fistulas, hemorrhoids)***	☐ Lecture	☐ Small Group
	☐ Online	☐ Self-directed
☐ ***Anorectal pain***	☐ Lecture	☐ Small Group
	☐ Online	☐ Self-directed
☐ ***Appendicitis***	☐ Lecture	☐ Small Group
	☐ Online	☐ Self-directed
☐ ***Ascites***	☐ Lecture	☐ Small Group
	☐ Online	☐ Self-directed
☐ ***Bowel cancer***	☐ Lecture	☐ Small Group
	☐ Online	☐ Self-directed
☐ ***Bowel dilation / obstruction***	☐ Lecture	☐ Small Group
	☐ Online	☐ Self-directed
☐ ***Celiac disease***	☐ Lecture	☐ Small Group
	☐ Online	☐ Self-directed
☐ ***Chronic abdominal pain***	☐ Lecture	☐ Small Group
	☐ Online	☐ Self-directed
☐ ***Chronic diarrhea***	☐ Lecture	☐ Small Group
	☐ Online	☐ Self-directed
☐ ***Diverticular disease***	☐ Lecture	☐ Small Group
	☐ Online	☐ Self-directed
☐ ***Dysmenorrhea***	☐ Lecture	☐ Small Group
	☐ Online	☐ Self-directed

- ☐ ***Dysphagia*** — ☐ Lecture ☐ Small Group ☐ Online ☐ Self-directed
- ☐ ***Esophageal cancer*** — ☐ Lecture ☐ Small Group ☐ Online ☐ Self-directed
- ☐ ***Esophageal motility disorders*** — ☐ Lecture ☐ Small Group ☐ Online ☐ Self-directed
- ☐ ***Fecal incontinence*** — ☐ Lecture ☐ Small Group ☐ Online ☐ Self-directed
- ☐ ***Food allergy / intolerance*** — ☐ Lecture ☐ Small Group ☐ Online ☐ Self-directed
- ☐ ***Gallstones*** — ☐ Lecture ☐ Small Group ☐ Online ☐ Self-directed
- ☐ ***Gastric cancer*** — ☐ Lecture ☐ Small Group ☐ Online ☐ Self-directed
- ☐ ***Gastrointestinal tumours*** — ☐ Lecture ☐ Small Group ☐ Online ☐ Self-directed
- ☐ ***GERD*** — ☐ Lecture ☐ Small Group ☐ Online ☐ Self-directed
- ☐ ***Hepatomegaly / hepatology*** — ☐ Lecture ☐ Small Group ☐ Online ☐ Self-directed
- ☐ ***Hernias*** — ☐ Lecture ☐ Small Group ☐ Online ☐ Self-directed
- ☐ ***Inflammatory bowel disease*** — ☐ Lecture ☐ Small Group ☐ Online ☐ Self-directed
- ☐ ***Irritable bowel syndrome*** — ☐ Lecture ☐ Small Group ☐ Online ☐ Self-directed
- ☐ ***Jaundice*** — ☐ Lecture ☐ Small Group ☐ Online ☐ Self-directed
- ☐ ***Liver cancer*** — ☐ Lecture ☐ Small Group ☐ Online ☐ Self-directed

☐ ***Lower GI bleeding***	☐ Lecture<br>☐ Online	☐ Small Group<br>☐ Self-directed
☐ ***Malabsorption***	☐ Lecture<br>☐ Online	☐ Small Group<br>☐ Self-directed
☐ ***Metabolic liver disease (i.e. NASH, Wilson's, hemochromatosis)***	☐ Lecture<br>☐ Online	☐ Small Group<br>☐ Self-directed
☐ ***Nausea / vomiting***	☐ Lecture<br>☐ Online	☐ Small Group<br>☐ Self-directed
☐ ***Nutritional support***	☐ Lecture<br>☐ Online	☐ Small Group<br>☐ Self-directed
☐ ***Obesity / bariatric surgery***	☐ Lecture<br>☐ Online	☐ Small Group<br>☐ Self-directed
☐ ***Pancreatic cancer***	☐ Lecture<br>☐ Online	☐ Small Group<br>☐ Self-directed
☐ ***Pancreatitis***	☐ Lecture<br>☐ Online	☐ Small Group<br>☐ Self-directed
☐ ***Pediatric constipation***	☐ Lecture<br>☐ Online	☐ Small Group<br>☐ Self-directed
☐ ***Pediatric diarrhea***	☐ Lecture<br>☐ Online	☐ Small Group<br>☐ Self-directed
☐ ***Peptic ulcer disease***	☐ Lecture<br>☐ Online	☐ Small Group<br>☐ Self-directed
☐ ***Pneumonia***	☐ Lecture<br>☐ Online	☐ Small Group<br>☐ Self-directed
☐ ***Splenomegaly***	☐ Lecture<br>☐ Online	☐ Small Group<br>☐ Self-directed
☐ ***Upper GI bleeding***	☐ Lecture<br>☐ Online	☐ Small Group<br>☐ Self-directed
☐ ***Viral hepatitis***	☐ Lecture<br>☐ Online	☐ Small Group<br>☐ Self-directed

13. Which of the following CanMEDs roles are *specifically* taught in your undergraduate Gastroenterology course?

- ☐ Medical expert
- ☐ Professional
- ☐ Manager
- ☐ Health Advocate
- ☐ Communicator
- ☐ Collaborator
- ☐ Scholar

14. Do you use a computer-based curriculum management system (i.e. One45, Medsys, etc.)?

- ○ No
- ○ Yes (please specify): ______

15. Do you use interactive web tools for teaching?

- ○ No
- ○ Yes (please describe) ______

16. Which of the following resources do you provide electronically to your students (choose all that apply)?

- ☐ Vodcasts
- ☐ Podcasts
- ☐ Powerpoint slides
- ☐ Other ______

17. Do you use social media for teaching (choose all that apply)?

- ☐ No
- ☐ Facebook
- ☐ Twitter
- ☐ Blogs
- ☐ Other ______

18. How are students assessed / evaluated in your Gastroenterology course (choose all that apply)?

- ☐ Interim quizzes / exam
- ☐ Final / summative exam
- ☐ Written assignments
- ☐ OSCE
- ☐ No formal assessment
- ☐ Other ______

19. Which of the following types of exam questions are used in your Gastroenterology course (choose all that apply)?

- ☐ Multiple choice
- ☐ Short answer
- ☐ Long answer / essay
- ☐ Computer-based examination
- ☐ Written examination
- ☐ Other ____________________

Thank you for completing this survey! Please indicate if the investigator may contact you for further information on this topic:

- ○ Yes
- ○ No

E-mail Address:

____

Is there anyone else involved in the curriculum that we may contact with this questionnaire / further questions?

- ○ Yes
- ○ No

Name:

____

E-mail Address:

____
